# Dosimetry and pharmacokinetics of [^177^Lu]Lu-satoreotide tetraxetan in patients with progressive neuroendocrine tumours

**DOI:** 10.1007/s00259-024-06682-1

**Published:** 2024-03-26

**Authors:** Seval Beykan Schürrle, Uta Eberlein, Catherine Ansquer, Jean-Mathieu Beauregard, Lucie Durand-Gasselin, Henning Grønbæk, Alexander Haug, Rodney J. Hicks, Nat P. Lenzo, Shaunak Navalkissoor, Guillaume P. Nicolas, Ben Pais, Magali Volteau, Damian Wild, Alexander McEwan, Michael Lassmann

**Affiliations:** 1 Department of Nuclear Medicine, University Hospital Würzburg, Würzburg, Germany; 2 CHU Nantes, Nantes Université, Médecine Nucléaire, Nantes, France; 3 Department of Medical Imaging, CHU de Québec – Université Laval, Québec City, QC Canada; 4 Ipsen Innovation, Les Ulis, France; 5 Department of Hepatology & Gastroenterology, ENETS Centre of Excellence, Aarhus University Hospital and Clinical Institute, Aarhus University, Aarhus, Denmark; 6 Department of Radiology and Nuclear Medicine, Medical University of Vienna, Vienna, Austria; 7 Department of Medicine, St Vincent’s Hospital, The University of Melbourne, Melbourne, VIC Australia; 8 Department of Medicine, Central Clinical School, the Alfred Hospital, Monash University, Melbourne, VIC Australia; 9 GenesisCare, East Fremantle, WA Australia; 10 Department of Medicine, Curtin University, Perth, WA Australia; 11 Neuroendocrine Tumour Unit, ENETS Centre of Excellence, Royal Free London NHS Foundation Trust, London, UK; 12 Division of Nuclear Medicine, ENETS Centre of Excellence, University Hospital Basel, Basel, Switzerland; 13 SRT-Biomedical B.V, Soest, Netherlands; 14 Ariceum Therapeutics GmbH, Berlin, Germany

**Keywords:** [^177^Lu]Lu-satoreotide tetraxetan, Somatostatin receptor antagonist, Neuroendocrine tumours, Dosimetry, Pharmacokinetics, Systemic radionuclide therapy

## Abstract

**Purpose:**

To evaluate the dosimetry and pharmacokinetics of the novel radiolabelled somatostatin receptor antagonist [^177^Lu]Lu-satoreotide tetraxetan in patients with advanced neuroendocrine tumours (NETs).

**Methods:**

This study was part of a phase I/II trial of [^177^Lu]Lu-satoreotide tetraxetan, administered at a median cumulative activity of 13.0 GBq over three planned cycles (median activity/cycle: 4.5 GBq), in 40 patients with progressive NETs. Organ absorbed doses were monitored at each cycle using patient-specific dosimetry; the cumulative absorbed-dose limits were set at 23.0 Gy for the kidneys and 1.5 Gy for bone marrow. Absorbed dose coefficients (ADCs) were calculated using both patient-specific and model-based dosimetry for some patients.

**Results:**

In all evaluated organs, maximum [^177^Lu]Lu-satoreotide tetraxetan uptake was observed at the first imaging timepoint (4 h after injection), followed by an exponential decrease. Kidneys were the main route of elimination, with a cumulative excretion of 57–66% within 48 h following the first treatment cycle. At the first treatment cycle, [^177^Lu]Lu-satoreotide tetraxetan showed a median terminal blood half-life of 127 h and median ADCs of [^177^Lu]Lu-satoreotide tetraxetan were 5.0 Gy/GBq in tumours, 0.1 Gy/GBq in the bone marrow, 0.9 Gy/GBq in kidneys, 0.2 Gy/GBq in the liver and 0.8 Gy/GBq in the spleen. Using image-based dosimetry, the bone marrow and kidneys received median cumulative absorbed doses of 1.1 and 10.8 Gy, respectively, after three cycles.

**Conclusion:**

[^177^Lu]Lu-satoreotide tetraxetan showed a favourable dosimetry profile, with high and prolonged tumour uptake, supporting its acceptable safety profile and promising efficacy.

**Trial registration:**

NCT02592707. Registered October 30, 2015.

**Supplementary Information:**

The online version contains supplementary material available at 10.1007/s00259-024-06682-1.

## Introduction

Radioligand therapy (RLT), including peptide receptor radionuclide therapy (PRRT), has evolved in recent decades. Multiple clinical trials have shown benefits of RLT, with an acceptable safety profile in the treatment of various cancers, including neuroendocrine tumours (NETs) [1–3], metastatic castration-resistant prostate cancer [4–6], advanced thyroid cancer [7], and pheochromocytoma and paraganglioma [8]. However, there are still challenges in defining the optimal activity regimen, hindering the full potential of RLT. Most clinical studies rely on cumulative absorbed dose thresholds, which are usually set at 23.0 Gy for the kidneys and 2.0 Gy for the bone marrow based on data from external beam radiotherapy [9–11]. Specific optimum thresholds for RLT (in which the quality and time-frame of radiation delivery vary markedly from conventional radiotherapy) are still to be defined.

Nevertheless, a growing body of evidence suggests that dosimetry can play a major role in clinical trials. In patients receiving RLT, estimation of absorbed doses may be valuable in determining dose-response relationships and predicting adverse outcomes associated with radiation exposure [12]. For example, in a cohort of 46 patients with advanced NETs treated with [^177^Lu]Lu-DOTA-TATE, a significant correlation was shown between the bone marrow absorbed dose and decreased platelet counts, regardless of the dosimetry method used [13]. In a cohort of 24 patients with pancreatic NETs treated with repeated cycles of [^177^Lu]Lu-DOTA-TATE at 8-week intervals, a significant correlation was shown between the tumour absorbed doses and tumour reduction [14]. In a retrospective analysis of a larger cohort of patients with gastroenteropancreatic (GEP)-NETs treated with [^177^Lu]Lu-DOTA-TATE, absorbed dose to tumours was shown to be predictive of radiologic response [15]. Absorbed dose estimation is therefore critical to the development and assessment of novel RLT.

Whilst PRRT with somatostatin receptor (SSTR) agonists is a well-established treatment for the management of advanced NETs [3, 16–18], radiolabelled SSTR antagonists may further improve responses, with previous studies demonstrating higher uptake and longer retention in tumours compared with SSTR agonists [16, 19, 20]. A novel radiolabelled SSTR antagonist, [^177^Lu]Lu-satoreotide tetraxetan (also known as [^177^Lu]^177^Lu-SSO110, [^177^Lu]^177^Lu-IPN01072, [^177^Lu]^177^Lu-OPS201 or [^177^Lu]^177^Lu-DOTA-JR11), has recently shown a favourable pharmacokinetic and biodistribution profile, as well as high metabolic stability in patients with NETs [21, 22]. In a preliminary clinical study of four patients with progressive NETs, [^177^Lu]Lu-satoreotide tetraxetan administered at a single mean activity of 1.0 GBq demonstrated, on average, a three-fold higher tumour absorbed dose and a two-fold higher tumour-to-kidney absorbed dose ratio compared with [^177^Lu]Lu-DOTA-TATE administered at a single mean activity of 1.1 GBq [22]. Similarly, in a subsequent phase I study of 20 patients with heavily pre-treated NETs, [^177^Lu]Lu-satoreotide tetraxetan, administered with an activity of 2.5–7.9 GBq per cycle for up to two cycles, showed high uptake in all known disease sites, with low uptake in other organs [23].

Here, we fully explore the biodistribution and dosimetry of [^177^Lu]Lu-satoreotide tetraxetan in a dosage-finding, phase I/II study (NCT02592707; registered October 30, 2015) of 40 patients with progressive, SSTR-positive NETs treated with [^177^Lu]Lu-satoreotide tetraxetan. A plain language summary of this publication can be found in the Supplementary Information. In the primary publication of this trial, we reported that [^177^Lu]Lu-satoreotide tetraxetan showed an acceptable safety profile, with no reported grade 3/4 kidney toxicity and the most common grade 3/4 treatment-related toxicities being lymphopenia, thrombocytopenia, and neutropenia, each occurring in 7.5% of patients [24]. The disease control rate at 12 months was 94.7%, and median progression-free survival based on independent central review was non-calculable at the time of analysis [24].

## Methods

### Patients and treatment protocol

Patients with pre-treated, unresectable, SSTR-positive NETs were enrolled across Australia, Europe, and Canada in this multicentre, multinational, open-label, non-randomised, phase I/II study. The study was approved by all relevant ethical committees, and patients gave written informed consent. Although the study was terminated early for practical reasons, all patients had completed treatment and most have been transferred to the long-term, five-year safety follow-up study.

**Fig. 1:**
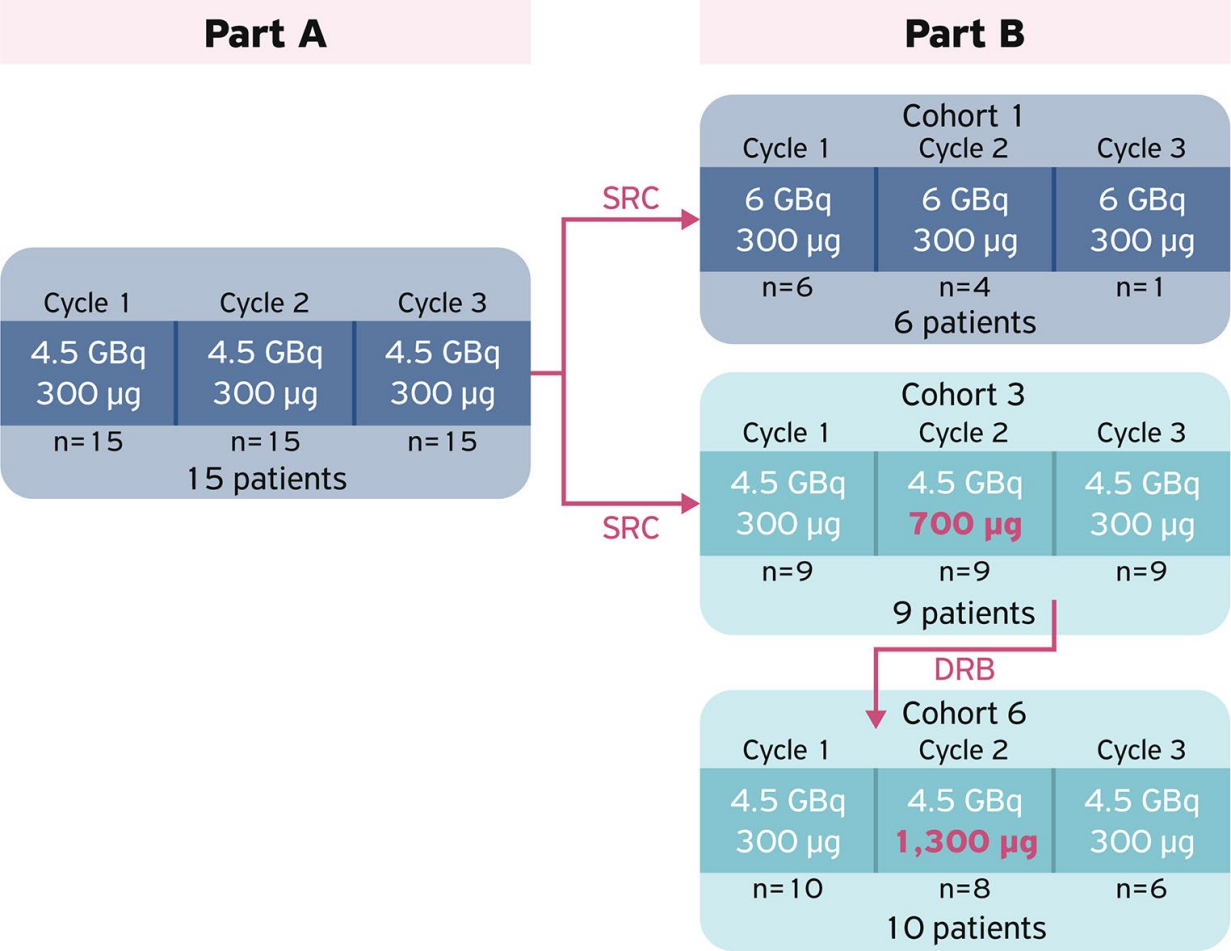
Administered activity and peptide amounts of [^177^Lu]Lu-satoreotide tetraxetan in cycles 1 to 3 of part A and part B of the study [24]. Note that four patients in cohort 3 and two patients in cohort 6 received additional treatment cycles. The study had a SRC (part **A**) and a DRB (part **B**). During part B, each escalation cohort (whether of administered activity or peptide amount) was evaluated by the DRB. The DRB recommended the cohort 1 administered activity be reduced from 6.0 GBq to 4.5 GBq due to safety concerns, and therefore the planned cohorts 2, 4, 5, 7 and 8, which would have included administered activities of more than 4.5 GBq, were not performed. Of the 40 included patients, 36 underwent dosimetry evaluation. Among them, 33 patients were treated with a [^177^Lu]Lu-satoreotide tetraxetan cycle of 4.5 GBq (part A: *N* = 11 [cycles 1–3]; part B: *N* = 22 [cycles 1–3 in cohort 1, cycles 1–3 in cohort 3, cycles 1–3 in cohort 6]) and three were treated with a [^177^Lu]Lu-satoreotide tetraxetan cycle of 6 GBq (cycle 1 of cohort 1 in part B). DRB: Data Review Board; SRC: Safety Review Committee

The full study design has been recently published [24]. The study was conducted in two parts for safety reasons (Fig. 1). Part A consisted of 15 patients who were administered three planned cycles of [^177^Lu]Lu-satoreotide tetraxetan at an activity of 4.5 GBq (± 10%) and a peptide mass dose of 300 µg (± 50) per cycle, resulting in a median cumulative administered activity of 13.1 GBq (range: 10.3–13.5 GBq). The median cumulative administered activity at each cycle is summarised in Table S1. Part B consisted of 25 patients divided into three cohorts (cohort 1, *N* = 6; cohort 3, *N* = 9; cohort 6, *N* = 10), and was initiated after safety and dosimetry data from part A were evaluated by a safety review committee. Part B investigated different activities (4.5 or 6.0 GBq per cycle) and peptide mass doses (300, 700 or 1,300 µg per cycle) of [^177^Lu]Lu-satoreotide tetraxetan, with activity escalation evaluated by a data review board (DRB). Cohort 1 received three cycles at a peptide amount of 300 µg (± 50) and administered activity of 6.0 GBq (± 10%). However, the activity was reduced to 4.5 GBq after three patients received treatment at 6.0 GBq; bone marrow absorbed dose exceeded the upper threshold (1.5 Gy) in two of these patients and one patient developed a grade 3 thrombocytopenia. Cohorts 3 and 6 investigated escalating peptide mass doses. Both cohort 3 and cohort 6 started with 300 µg (± 50) for cycle 1, increasing to 700 µg (± 150) in cohort 3 and 1,300 µg (± 200) in cohort 6 for cycle 2, then repeating the 300 µg (± 50) dosing for cycle 3, each at an administered activity of 4.5 GBq (± 10%). Overall, the median cumulative administered activity of [^177^Lu]Lu-satoreotide tetraxetan in part B was 12.9 GBq (range: 4.2–20.8 GBq), and the median number of therapy cycles was three (range: 1–5).

[^177^Lu]Lu-satoreotide tetraxetan was administered on day one of each cycle as an intravenous infusion over 120 min. The time interval between each treatment cycle was 8–12 weeks. In both parts A and B, the administered activity and the number of treatment cycles of [^177^Lu]Lu-satoreotide tetraxetan could be adapted based on kidney and bone marrow dosimetry results over prior cycles to ensure compliance with the cumulative absorbed-dose limits (23.0 Gy for the kidneys and 1.5 Gy for the bone marrow). Compared to the 2.0 Gy bone marrow absorbed-dose limit recommended by the International Commission on Radiological Protection [10], this conservative threshold of 1.5 Gy was chosen based on safety results of an earlier phase I study of [^177^Lu]Lu-satoreotide tetraxetan [23].

Renal absorbed dose, potentially the treatment-limiting factor of PRRT, can be effectively reduced by the concomitant administration of cationic amino acids [25]. For kidney protection, an amino acid infusion (arginine and lysine solution at a concentration of 1.25% w/v in 2 L saline) was given concomitant to the PRRT administration over 4 h, starting 30 to 60 min before the infusion of [^177^Lu]Lu-satoreotide tetraxetan. The infusion time could be extended to 6 h at the discretion of the investigator in case of technical infusion problems, interruption of infusion due to adverse events (AEs), or patients’ intolerance of the high-volume load in a short time.

### Image acquisition

In both parts A and B of the study, patients underwent planar whole-body imaging at 4 h and 1, 2, 3, and 6 days after each [^177^Lu]Lu-satoreotide tetraxetan treatment cycle. Patients in part A also underwent single-photon emission computed tomography/computed tomography (SPECT/CT) at 24 h after each [^177^Lu]Lu-satoreotide tetraxetan cycle. Those in part B underwent SPECT/CT, immediately before or after planar scintigraphy, at 4 h, and 1, 2, 3, and 6 days after each [^177^Lu]Lu-satoreotide tetraxetan administration. Full details of the image acquisition methodology are provided in the Supplementary Information.

### Blood and urine samples

To generate pharmacokinetic data, blood samples (2 mL) were taken at the stop of infusion (0 min) and 5, 30, and 60 min, 4 h, and 1, 2, 3, and 6 days after the end of each [^177^Lu]Lu-satoreotide tetraxetan administration. Urine samples were collected over three intervals within 48 h (0–6 h, 6–24 h, and 24–48 h in part A; 0–4 h, 4–24 h, and 24–48 h in part B) following the first treatment cycle of [^177^Lu]Lu-satoreotide tetraxetan. Total activity concentrations in whole blood and urine were determined locally, using a gamma counter calibrated for lutetium-177. In two selected study centres, additional blood samples (2 mL) were collected at 1 min, 4 h, and 1, 2, 3, and 6 days after each [^177^Lu]Lu-satoreotide tetraxetan administration to evaluate potential radioactive metabolites in plasma.

### Delineation and [^177^Lu]Lu-satoreotide tetraxetan activity quantification

Delineation of tumour volumes was performed on anatomical SPECT/CT images after each [^177^Lu]Lu-satoreotide tetraxetan administration. Tumours with a longest diameter < 2 cm were not analysed due to the difficulty in correcting for partial volume effects caused by the limited spatial resolution of the SPECT/CT systems. For study inclusion in part A, one tumour lesion ≥ 2 cm must have been present for eligibility; in part B, two or more tumour lesions ≥ 2 cm must have been present, each with an uptake on SSTR imaging higher than that of normal liver parenchyma (target lesion on [^68^Ga]Ga-DOTA-TATE or -DOTA-TOC positron emission tomography: maximum standardised uptake value [SUV_max_] ≥ 2x the mean SUV [SUV_mean_] of liver background; or ^111^In-scintigraphy/SPECT: Krenning score 3 or 4 [uptake > normal liver, or uptake > spleen or kidneys, respectively [26]]).

Reconstruction parameters were set up to produce isotropic voxels of < 5 mm; voxels were continuous throughout the field of view. Anatomic coverage of the acquisition was determined according to the patients’ tumour locations on the planar whole-body scan acquisition with liver, both kidneys and spleen in the same bed position. For organs showing [^177^Lu]Lu-satoreotide tetraxetan uptake, regions of interest (ROIs)/volumes of interest (VOIs) were drawn manually over the whole organs of interest (whole body, left and right kidneys, liver, spleen, and bone marrow), using the NUKDOS software for planar whole-body scans and the SPECT/CT acquired in part A [27]. VOIs for the same organs were drawn for all SPECT/CT images acquired in part B. All ROIs/VOIs were then used for activity quantification. Time-integrated activity coefficients (TIACs) for a given source volume were computed by integrating the time-activity curves. Full details of the tumour delineation and activity quantification methodology are provided in the Supplementary Information.

### Image calibration

To ensure comparability of gamma counter results and image quantification of the SPECT/CT images at each study centre, three low-activity samples were centrally prepared and shipped to the study centres for calibration. Full details of the calibration process are provided in the Supplementary Information.

### Dosimetry

Patient-specific dosimetry calculations were performed after each treatment cycle in both parts A and B, using the absorbed dose calculation features of the NUKDOS software for image-based dosimetry calculations [27]. The NUKDOS software was chosen as it allowed for an integral approach to organ dosimetry (including image quantification, time-activity curve-fitting and dosimetry calculation). For the bone marrow, both image-based and blood-based calculations were performed in part A [28, 29]. In part B, only image-based bone marrow dosimetry was performed [28, 29]. Organ Level INternal Dose Assessment/EXponential Modelling (OLINDA/EXM) version 1.0 software, the most widely used version at the time of the study, was used to perform model-based dosimetry calculations [30], which were only performed after the first [^177^Lu]Lu-satoreotide tetraxetan cycle for patients in part B. To generate organ-specific absorbed doses, TIACs were used as input data. Full details on OLINDA/EXM software settings and organ-specific TIAC calculation methods are provided in the Supplementary Information.

### Statistical analysis

All statistical analyses were performed using SAS version 9.2 or 9.3 (SAS Institute, Cary, NC). The per-protocol dosimetry analysis set (*N* = 36) was used for all statistical analyses, defined as all patients who received at least one cycle of [^177^Lu]Lu-satoreotide tetraxetan treatment with at least one post-baseline dosimetry assessment and no major protocol violations affecting dosimetry variables. For Cohorts 3 and 6 in Part B, to allow significant power to detect a difference between two peptide doses within the same cohort, a total sample size of 8 was calculated. Assuming 30% standard deviation of the paired difference, this provides 80% power to detect a 2-fold-change, and 99% power to detect a 3-fold change, at a 2-sided alpha of 0.05. Statistical analysis was descriptive: variables were expressed as mean ± standard deviation (SD), median, and range. Missing values were not replaced.

## Results

### Patients

Overall, 40 patients (21 male and 19 female; median, range age: 62.5 years, 27–82 years) were enrolled in this study. 29 (72.5%), 7 (17.5%), and 4 (10.0%) were initially diagnosed with GEP-NETs, lung NETs, and pheochromocytoma and paraganglioma, respectively (Table 1). Of the 40 included patients, four were excluded from the per-protocol dosimetry analysis set due to issues in the SPECT/CT calibration process impacting all treatment cycles, which was classified as a major protocol deviation. Therefore, data from these patients was not included in this analysis due to uncertainty around the validity of the calibration and 36 successfully underwent dosimetry evaluation. Among them, 33 were treated with a [^177^Lu]Lu-satoreotide tetraxetan cycle of 4.5 GBq (part A: *N* = 11 [cycles 1–3]; part B: *N* = 22 [cycles 1–3 in cohort 1, cycles 1–3 in cohort 3, cycles 1–3 in cohort 6]) and three were treated with a [^177^Lu]Lu-satoreotide tetraxetan cycle of 6 GBq (cycle 1 of cohort 1 in part B). Of those who received [^177^Lu]Lu-satoreotide tetraxetan, 25 patients (69.4%) completed the three planned cycles of therapy and, of these, five and one were able to receive one and two additional treatment cycles, respectively. Reasons for patients not completing the planned three cycles included AEs, disease progression and exceeding the cumulative bone marrow dose limit. Median cumulative activity was 13.0 GBq (range: 4.2–20.8 GBq) over the three planned cycles.

**Table 1:** Baseline characteristics of the overall study population

Characteristic	Total (*N* = 40)
Age (years)	
Median (range) Mean ± SD	62.5 (27–82) 59.9 ± 13.5
Sex	
Male Female	21 (52.5) 19 (47.5)
Time since initial diagnosis (months)	
Median (range)	45.2 (5.6–157.9)
Time from last relapse to screening (months)	
Median (range)	1.1 (–0.3–5.7)
Karnofsky performance status	
80 90 100	1 (2.5) 27 (67.5) 12 (30.0)
Initial diagnosis	
Gastroenteropancreatic NET Lung NET Pheochromocytoma/paraganglioma	29 (72.5) 7 (17.5) 4 (10.0)
Primary tumour type	
Gastrointestinal	19 (47.5)
Pancreas	9 (22.5)
Lung	7 (17.5)
Paraganglioma and pheochromocytoma	4 (10.0)
Unknown	1 (2.5)
Tumour grade	
1 2 Unknown	9 (22.5) 23 (57.5) 8 (20.0)
Ki-67 proliferation index (in %)	
Median (range) Mean ± SD Not evaluable	5.0 (1.0–20.0) 6.6 ± 5.1 4 (10.0)
Prior treatments	
Somatostatin analogues Surgery Chemotherapy Radiotherapy	30 (75.0) 29 (72.5) 10 (25.0) 8 (20.0)

Data are presented as n (%), unless otherwise specified. NET: neuroendocrine tumour; SD: standard deviation

### Biodistribution and dosimetry

#### Time-activity curves

**Fig. 2:**
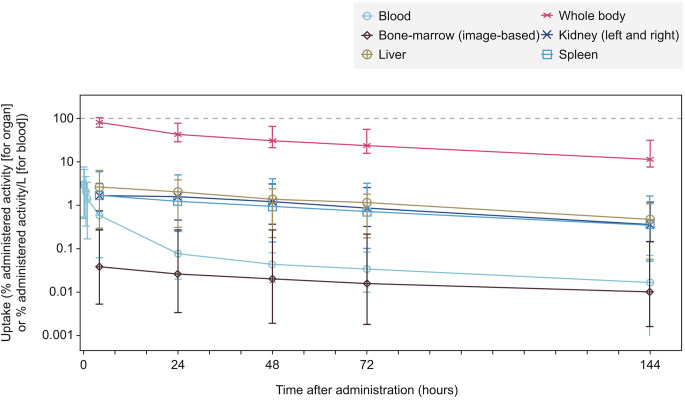
Uptake of [^177^Lu]Lu-satoreotide tetraxetan after administration of the first treatment cycle in parts A and B. Per protocol dosimetry analysis set (*N* = 36). Data are presented as median (range), in percentage of administered activity or % of administered activity/L for blood uptake. Lines are not directly related to the fit of the data and should be used as a guide only. The grey dashed line represents 100% uptake

For the first [^177^Lu]Lu-satoreotide tetraxetan treatment cycle, time-activity curves showed that there was a similar and prolonged uptake pattern of [^177^Lu]Lu-satoreotide tetraxetan in the whole body (median of 11.5% administered activity at 6 days) and in evaluated organs (Fig. 2). In all evaluated organs, the maximum [^177^Lu]Lu-satoreotide tetraxetan uptake was observed at the first imaging timepoint (4 h after injection), followed by an exponential decrease.

### Absorbed dose coefficients

Cycle 1 and cycle 3 ADCs in tumours and evaluated organs are presented in Table 2. [^177^Lu]Lu-satoreotide tetraxetan exhibited higher absorbed dose coefficients (ADCs) in tumours compared with evaluated organs across cycles 1 and 3, regardless of cohort (Table 2). The median ADC of [^177^Lu]Lu-satoreotide tetraxetan in tumours was 5.0 Gy/GBq (range: 0.4–83.3 Gy/GBq) after administration of the first treatment cycle (part A median [range]: 2.6 [0.4–14.3] Gy/GBq; part B median [range]: 7.9 [1.9–83.3] Gy/GBq). Cycle 1 ADC results are also illustrated in Fig. **S1**.

**Table 2:** Absorbed dose coefficients of [^177^Lu]Lu-satoreotide tetraxetan in tumours and evaluated organs after treatment cycles 1 and 3, and their ratios

			Tumours (*N* = 36)	Bone marrow* (*N* = 36)	Kidneys (*N* = 36)	Liver (*N* = 36)	Spleen (*N* = 36)
**Cycle 1** **ADC**, **Gy/GBq**	Part A and B	n	33	29	35	8	27
		Median (Range)	5.0 (0.4–83.3)	0.1 (0.0–0.6)	0.9 (0.4–1.9)	0.2 (0.1–0.3)	0.8 (0.2–1.8)
	Part A	n	11	11	11	7	9
		Median (Range)	2.6 (0.4–14.3)	0.1 (0.0–0.2)	1.1 (0.5–1.9)	0.2 (0.1–0.3)	0.8 (0.2–1.8)
	Part B Cohort 1	n	5	6	6	1	6
		Median (Range)	3.9 (1.9–83.3)	0.1 (0.1–0.6)	0.7 (0.6–1.0)	0.2 (0.2–0.2)	1.0 (0.5–1.1)
	Part B Cohort 3	n	9	6	9	0	6
		Median (Range)	6.8 (2.1–28.2)	0.1 (0.1–0.1)	0.9 (0.5–1.2)	NR	1.0 (0.2–1.5)
	Part B Cohort 6	n	8	6	9	0	6
		Median (Range)	13.5 (2.2–81.0)	0.1 (0.0–0.2)	0.8 (0.4–1.1)	NR	0.8 (0.4–1.0)
**Cycle 3 ** **ADC, ** **Gy/GBq**	Part A and B	n	24	21	26	7	19
		Median (Range)	4.0 (0.3–70.3)	0.1 (0.0–0.2)	0.8 (0.4–1.8)	0.2 (0.1–0.4)	0.7 (0.3–1.5)
	Part A	n	11	11	11	7	9
		Median (Range)	1.7 (0.3–5.0)	0.1 (0.0–0.2)	1.0 (0.7–1.8)	0.2 (0.1–0.4)	0.5 (0.3–1.0)
	Part B Cohort 1	n	0	1	1	0	1
		Median (Range)	NR	0.1 (0.1–0.1)	0.7 (0.7–0.7)	NR	0.5 (0.5–0.5)
	Part B Cohort 3	n	8	6	8	0	5
		Median (Range)	5.5 (2.6–16.8)	0.1 (0.0–0.1)	0.8 (0.4–1.2)	NR	0.8 (0.3–1.1)
	Part B Cohort 6	n	5	3	6	0	4
		Median (Range)	7.7 (3.2–70.3)	0.1 (0.0–0.1)	0.8 (0.7–1.2)	NR	0.8 (0.7–1.5)
**Ratio of** **Cycle 3/** **Cycle 1** **ADCs, %**	Part A and B	n	23	21	25	7	18
		Median (Range)	78.6 (16.0–195)	105 (22.4–400)	98.6 (59.1–198)	88.5 (43.8–132)	83.2 (40.9–231)
	Part A	n	11	11	11	7	9
		Median (Range)	76.7 (16.0–195)	101 (22.4–302)	106 (59.1–139)	88.5 (43.8–132)	61.5 (40.9–231)
	Part B Cohort 1	n	0	1	1	0	1
		Median (Range)	NR	64.3 (64.3–64.3)	111 (111–111)	NR	82.3 (82.3–82.3)
	Part B Cohort 3	n	8	6	8	0	5
		Median (Range)	80.5 (44.1–152)	106 (36.4–138)	94.1 (78.9–146)	NR	87.8 (76.0–152)
	Part B Cohort 6	n	4	3	5	0	3
		Median (Range)	79.0 (28.6–114)	200 (175–400)	143 (88.8–198)	NR	185 (84.2–192)

Per protocol dosimetry analysis set (*N* = 36). *For the bone marrow, image-based dosimetry is applied. When applying blood-based dosimetry (*N* = 11), the median absorbed dose coefficient for the bone marrow after cycle 1 was 0.03 Gy/GBq (range, 0.01–0.04 Gy/GBq). ADC: absorbed dose coefficient; NR: not reported

Median ADCs of [^177^Lu]Lu-satoreotide tetraxetan in tumours were higher in patients initially diagnosed with GEP-NETs (5.8 Gy/GBq; range: 0.6–83.3 Gy/GBq) and lung NETs (5.1 Gy/GBq; range: 2.1–28.2 Gy/GBq), compared with pheochromocytoma and paraganglioma (2.1 Gy/GBq; range: 0.4–4.9 Gy/GBq) after administration of the first treatment cycle (Table S2).

ADCs in tumours decreased over the course of treatment, as shown by a median ADC of 4.0 Gy/GBq (range: 0.3–70.3 Gy/GBq) after cycle 3 and a median ratio of ADCs between cycle 3 and cycle 1 in tumours of 78.6% (range: 16.0–195%). This phenomenon was consistently observed in each study cohort, with the median ADC ratio between cycle 3 and cycle 1 in tumours ranging from 76.7% (range: 16.0–195%) in part A to 80.5% (range: 44.1–152%) in cohort 3 of part B. There were limited observations (6.5%) of a median ADC ratio between cycle 3 and cycle 1 in tumours > 150%. These observations were only applicable to certain tumours in these patients, and were not consistently associated with a low ADC at cycle 1 (range: 0.6–4.1 Gy/GBq). Similarly, ADCs tended to decrease over the course of treatment in the spleen. For the kidneys, the bone marrow, and the liver (though liver data were available for seven patients only), limited differences in ADCs were observed between cycle 3 and cycle 1. Median ADC ratios > 150% between cycle 3 and cycle 1 in organs were observed in 23.8% of patients in the bone marrow, 8.3% of patients in the kidneys, 22.2% of patients in the spleen and no patients in the liver.

The median ratios of ADCs in evaluated organs and tumours between cycle 2 and cycles 1 and 3 are presented in Table 3. For the spleen, liver lesions, and all lesions, the median ratios of ADCs between cycle 2/cycle 1 and cycle 2/cycle 3 were consistently below 100% for cohorts 3 and 6, which received higher peptide mass doses at cycle 2.

**Table 3:** Ratios of absorbed dose coefficients of [^177^Lu]Lu-satoreotide tetraxetan in tumours and evaluated organs between Cycle 2 and Cycles 1 and 3

Location	Part A (4.5 GBq/cycle) *n* = 11				Part B Cohort 3 (4.5 GBq/cycle) *n* = 9				Part B Cohort 6 (4.5 GBq/cycle) *n* = 10			
	Cycle 2/Cycle 1 (300 vs. 300 µg)		Cycle2/Cycle 3 (300 vs. 300 µg)		Cycle 2/Cycle 1 (700 vs. 300 µg)		Cycle 2/Cycle 3 (700 vs. 300 µg)		Cycle 2/Cycle 1 (1300 vs. 300 µg)		Cycle 2/Cycle 3 (1300 vs. 300 µg)	
	n	Median (range)	n	Median (range)	n	Median (range)	n	Median (range)	n	Median (range)	n	Median (range)
Body	11	108 (38.3–142)	11	95.4 (69.5–119)	9	83.3 (52.8–100)	8	101 (78.7–132)	7	82.3 (48.3–97.6)	6	87.6 (72.7–102)
Bone marrow*	10	89.5 (54.7–152)	10	109 (23.9–297)	6	95.0 (36.4–143)	6	95.0 (60.0–125)	4	200 (80.0–240)	3	114 (50.0–120)
Kidney	11	116 (55.8–140)	11	101 (80.6–179)	9	80.8 (61.0–113)	8	82.0 (64.8–110)	7	92.5 (65.9–121)	6	72.4 (52.8–96.3)
Spleen	9	93.0 (50.0–270)	9	127 (81.1–334)	6	61.9 (45.9–108)	5	79.3 (61.2–90.8)	5	48.0 (44.3–94.4)	4	49.7 (49.3–52.6)
	**n/n-les**	**Median (range)**	**n/n-les**	**Median (range)**	**n/n-les**	**Median (range)**	**n/n-les**	**Median (range)**	**n/n-les**	**Median (range)**	**n/n- les**	**Median (range)**
All lesions	11/24	83.4 (28.6–156)	11/23	125 (73.3–343)	9/17	62.5 (27.6–95.7)	8/15	77.6 (52.4–121)	6/13	44.4 (24.7–76.5)	5/11	67.9 (40.6–104)
Liver lesions	9/16	91.8 (28.6–156)	9/16	127 (73.3–343)	7/10	70.4 (52.4–95.7)	6/9	68.9 (57.3–121)	4/9	48.4 (24.7–76.5)	4/9	67.9 (40.6–104)

Ratios are presented as %. Per protocol dosimetry analysis set. In part A, patients were administered 300 µg (± 50) per cycle at an administered activity of 4.5 GBq (± 10%). In part B, both cohort 3 and cohort 6 started with 300 µg (± 50) for cycle 1, increasing to 700 µg (± 150) in cohort 3 and 1,300 µg (± 200) in cohort 6 for cycle 2, then repeating the 300 µg (± 50) dosing for cycle 3, each at an administered activity of 4.5 GBq (± 10%). The median ratios of ADCs between cycle 2 and cycles 1 and 3 for cohort 1 of part B are not presented. *For the bone marrow, image-based dosimetry is applied. N-les: number of lesions

### Cumulative absorbed doses

**Fig. 3:**
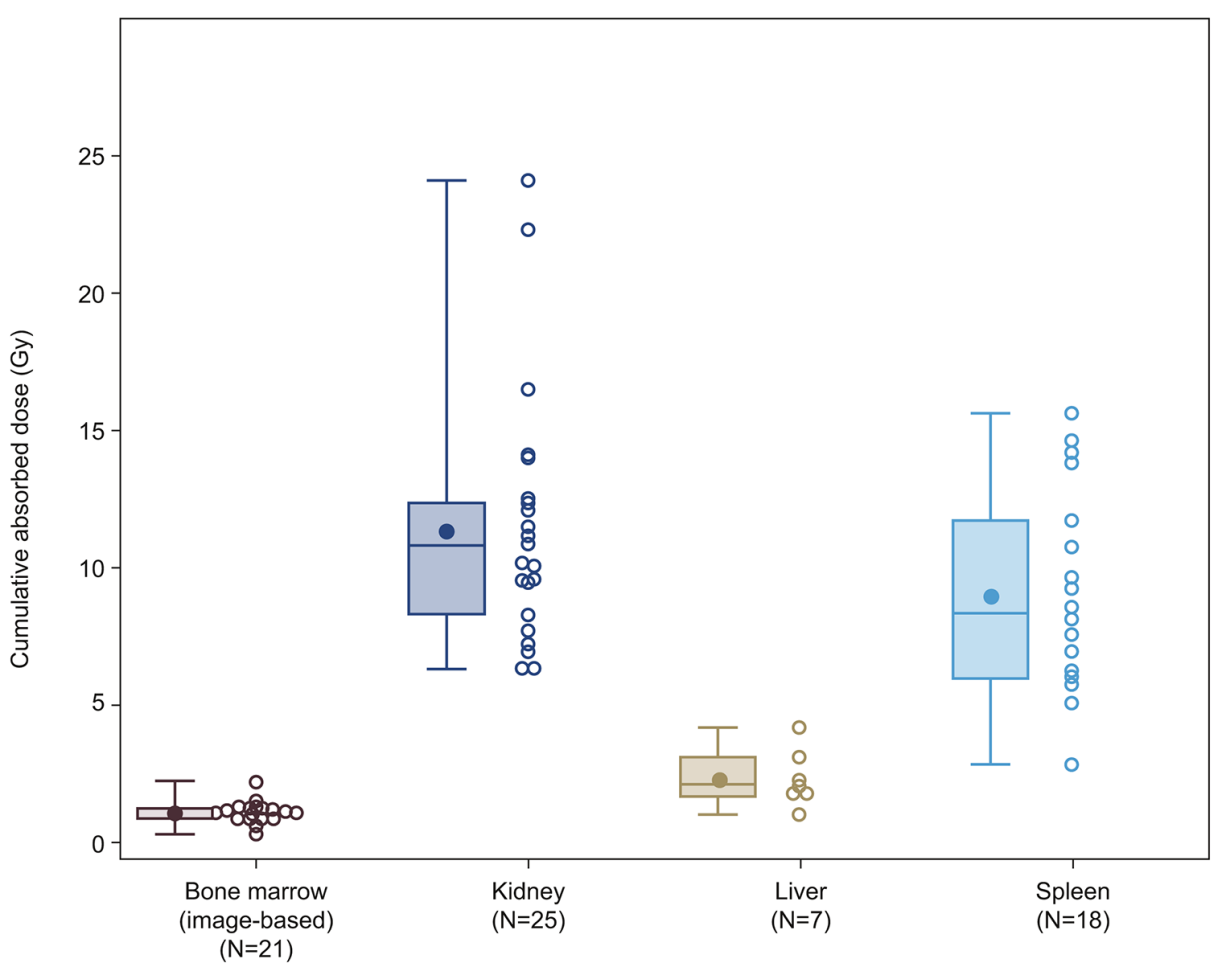
Cumulative absorbed doses of [^177^Lu]Lu-satoreotide tetraxetan in evaluated organs after administration of three [^177^Lu]Lu-satoreotide tetraxetan cycles in parts A and B. Per protocol dosimetry analysis set (*N* = 36). Data are presented as mean, median (minimum, Q1, Q2, Q3, maximum) for parts A and B in box plot; scatter plots represent individual patient data for parts A and B. Q1: quartile 1; Q2: quartile 2; Q3: quartile 3

The median cumulative absorbed doses (Gy) in all evaluated organs after treatment cycles 1–3 are illustrated in Fig. 3 and were below the pre-specified limits in the bone marrow and kidneys. Based on imaging in parts A and B, among patients who completed three cycles of [^177^Lu]Lu-satoreotide tetraxetan therapy (*n* = 21), the bone marrow received a median cumulative absorbed dose of 1.1 Gy (range: 0.3–2.2 Gy; pre-specified absorbed-dose limit: 1.5 Gy). Dosimetry monitoring in the bone marrow after administration of the first treatment cycle could not be achieved for seven patients. In part A, following administration of cycles 1–3 of [^177^Lu]Lu-satoreotide tetraxetan in 11 evaluated patients, the bone marrow blood-based method identified a lower cumulative absorbed dose (median: 0.3 Gy; range: 0.2–0.5 Gy) than the image-based method (median: 1.1 Gy; range: 0.6–2.2 Gy). In both parts A and B, the kidneys (both left and right) received a median cumulative absorbed dose of 10.8 Gy (range: 6.3–24.1 Gy) after cycles 1–3 (pre-specified limit: 23.0 Gy).

In three patients, the cumulative bone marrow absorbed dose exceeded the 1.5 Gy threshold during treatment cycles 1–3; two did not develop any acute haematological toxicity (one part B cohort 1 patient who received 1.9 Gy across two treatment cycles at 4.7 GBq, and one part A patient reaching 2.2 Gy who received three treatment cycles at 4.5 GBq, 4.4 GBq, and 4.3 GBq), while one patient developed grade 3 thrombocytopenia (a cohort 1 patient treated with one cycle at 5.9 GBq who received 3.6 Gy). The cumulative bone marrow absorbed dose exceeded the 1.5 Gy threshold in two additional patients after treatment cycle 4, reaching 1.5 and 1.8 Gy; one of these patients developed a grade 3 treatment-emergent AE (TEAE; grading determined by the US National Cancer Institute Common Terminology Criteria for AEs [NCI-CTCAE] version 5.0) coded as ‘lymphocyte count decreased’. The cumulative kidney absorbed dose also exceeded the 23.0 Gy threshold in one part A patient (reaching a mean cumulative absorbed dose of 24.1 Gy in the left and right kidneys), who had an estimated glomerular filtration rate below the normal limit at baseline and throughout the study and received three [^177^Lu]Lu-satoreotide tetraxetan treatment cycles each at 4.5 GBq. This patient did not develop any further deterioration of renal function during the seven months they were enrolled in the study and entered the long-term follow-up study.

Overall, dosimetry monitoring led to two patient withdrawals before cycle 3 (in cohort 1). Reductions in administered activity were deemed necessary for three patients (two in cohort 1 of part B before cycle 3, and one in cohort 3 at cycle 4).

Organ model-based dosimetry estimated using OLINDA/EXM, evaluated in 24 part B patients, is summarised in Table 4. Six patients were excluded from model-based dosimetry evaluations for the red bone marrow and the spleen due to lesions showing high uptake of [^177^Lu]Lu-satoreotide tetraxetan in the corresponding VOIs. The organs with the highest median ADCs by OLINDA/EXM were the kidneys (1.05 Gy/GBq; range: 0.52–1.46) and the spleen (0.65 Gy/GBq; range: 0.24–3.31). Besides the red bone marrow, for which a median cumulative absorbed dose of 1.47 Gy was calculated over three cycles of 4.5 GBq each, all other organs reached low cumulative absorbed doses compared to the limits identified by the International Commission on Radiological Protection [10].

**Table 4:** Dosimetry results calculated by OLINDA/EXM version 1.0 [30]

	ADC (Gy/GBq) (*N* = 24)*		Calculated absorbed dose for 3 cycles of 4.5 GBq each (Gy) (*N* = 24)*	
Organ	Median	Range	Median	Range
Adrenals	0.08	0.04–0.21	1.03	0.57–2.85
Brain	0.07	0.04–0.20	0.96	0.50–2.74
Breasts	0.07	0.04–0.20	0.95	0.52–2.70
Gallbladder wall	0.08	0.04–0.21	1.02	0.55–2.86
Heart wall	0.07	0.04–0.21	0.99	0.53–2.84
Kidneys	1.05	0.52–1.46	14.18	6.99–19.71
Liver	0.08	0.04–0.21	1.07	0.53–2.82
LLI wall	0.07	0.04–0.21	1.00	0.53–2.85
Lungs	0.07	0.04–0.21	0.98	0.52–2.79
Muscle	0.07	0.04–0.20	0.97	0.51–2.75
Osteogenic cells	0.30	0.14–0.67	3.96	1.92–9.07
Ovaries	0.07	0.04–0.21	1.00	0.53–2.85
Pancreas	0.08	0.04–0.21	1.05	0.57–2.88
Red marrow	0.11	0.07–0.38	1.47	0.95–5.17
Skin	0.07	0.04–0.20	0.93	0.49–2.67
Small intestine	0.07	0.04–0.21	1.00	0.54–2.84
Spleen	0.65	0.24–3.31	8.78	3.24–44.69
Stomach wall	0.07	0.04–0.21	1.01	0.54–2.84
Testes**	0.05	0.04–0.12	0.72	0.53–1.59
Thymus	0.07	0.04–0.21	0.98	0.51–2.79
Thyroid	0.07	0.04–0.20	0.96	0.51–2.75
ULI wall	0.07	0.04–0.21	1.01	0.54–2.85
UB wall	0.07	0.04–0.21	0.99	0.52–2.84
Uterus**	0.08	0.06–0.21	1.13	0.77–2.85
**Total body**	**0.08**	**0.05–0.21**	**1.08**	**0.62–2.84**

*For the red marrow and the spleen, *N* = 18. **The absorbed dose coefficients of testes and uterus were calculated for male (*N* = 13) and female (*N* = 11) patients separately. ADC: absorbed dose coefficient; LLI: lower large intestine; UB: urinary bladder; ULI: upper large intestine

No radioactive metabolites were observed in plasma samples. Full pharmacokinetics results from this study can be found in the Supplementary Information.

## Discussion

In this analysis of 36 patients with progressive, SSTR-positive NETs who received [^177^Lu]Lu-satoreotide tetraxetan at a median cumulative activity of 13.0 GBq over three planned cycles, [^177^Lu]Lu-satoreotide tetraxetan was associated with good tumour uptake and retention, as well as a favourable pharmacokinetic and dosimetric profile. These findings are consistent with previous studies of [^177^Lu]Lu-satoreotide tetraxetan in patients with NETs [22, 23].

The absorbed doses of [^177^Lu]Lu-satoreotide tetraxetan for the kidneys, liver and spleen were tolerable after three cycles of 4.5 GBq for most patients. Accordingly, we did not observe signs of non-haematologic toxicities after [^177^Lu]Lu-satoreotide tetraxetan treatment [24], consistent with other studies of ^177^Lu-labelled SSTR antagonists [22, 23, 31]. However, the organ ADCs observed were lower than in other studies of [^177^Lu]Lu-satoreotide tetraxetan [22, 23], and another novel radiolabelled SSTR antagonist, [^177^Lu]Lu-DOTA-LM3 [31]. Results from this study were more consistent with the NETTER-1 phase III dosimetry substudy, which enrolled 20 patients with metastatic midgut NETs treated with [^177^Lu]Lu-DOTA-TATE [32, 33]. The differences between organ absorbed doses in different studies may result in part from differences in patient cohorts and the adopted delineation and dosimetry techniques.

In the present study, we observed tumour ADCs of 5.0 Gy/GBq after cycle 1 and 4.0 Gy/GBq after cycle 3. These tumour ADCs are consistent with previously reported findings from a direct comparison of [^177^Lu]Lu-satoreotide tetraxetan and [^177^Lu]Lu-DOTA-TATE in the same tumours and patients in a cross-over design, which reported median tumour dose of [^177^Lu]Lu-satoreotide tetraxetan of 7.0 Gy/GBq (range: 4.2–29 Gy/GBq), 3.5 times greater than the median tumour dose of [^177^Lu]Lu-DOTA-TATE [22]. However, it should be noted that median tumour dose in this pilot study was calculated from a limited dataset of 13 lesions in 4 patients. In a systematic review of the dosimetry profile of [^177^Lu]Lu-DOTA-TATE, median absorbed dose to the tumour was found to be 4.6 Gy/GBq (range: 3.1–9.5 Gy/GBq) across 799 studies [34], similar to the tumour ADCs reported here following [^177^Lu]Lu-satoreotide tetraxetan treatment. However, comparison of tumour ADCs across studies may not be meaningful, due to the difference in methodology, patients and tumour types.

Indeed, our data show differences in ADCs for tumours of different types, with higher median ADCs for lesions in patients initially diagnosed with GEP-NETs and lung NETs compared with pheochromocytoma and paraganglioma; however, the numbers of patients with lung NETs and pheochromocytoma and paraganglioma were low. ADCs in tumours were shown to decrease over the course of treatment (Table 2), which may reflect depopulation of SSTR-expressing cells, reduced delivery or decreased retention of the treatment at the tumour sites, following treatment with PRRT. A recent study in patients with GEP-neuroendocrine neoplasms treated with [^177^Lu]Lu-DOTA-TATE reported similar findings, though in this study a simplified dosimetry protocol was used [15]. The observed large variations in tumour ADCs likely reflect the significant intra- and inter-patient heterogeneity that exists in SSTR expression. Future studies may explore how variations in ADCs in matched lesions correlate with clinical, radiological and biochemical response to treatment. Variability in SSTR expression and organ dosimetry may strengthen the case for individualised prescription of administered activity to maximise absorbed dose to the tumours.

The median ADC of [^177^Lu]Lu-satoreotide tetraxetan for the bone marrow using a blood-based approach was lower than reported in other [^177^Lu]Lu-satoreotide tetraxetan studies [22, 23]. The result is, however, similar to the mean blood-based radiation ADCs for the bone marrow reported for [^177^Lu]Lu-DOTA-TATE [32]. In a previous study in patients with NETs treated with [^177^Lu]Lu-DOTA-TATE, it was found that that image-based and blood-based bone marrow ADCs were up to 0.06 Gy/GBq and 0.02 Gy/GBq, respectively [35]; given this substantial difference, the authors encouraged the use of image based dosimetry to prevent underestimation of the radiation absorbed dose to the bone marrow [35]. Similarly, in our study, bone marrow absorbed dose was evaluated using both image- and blood-based calculations in part A; image-based dosimetry resulted in a higher cumulative absorbed dose than blood-based imaging (median 1.1 Gy/GBq versus 0.3 Gy/GBq, respectively), which is in line with several dosimetry studies of [^177^Lu]Lu-DOTA-TATE [13, 35, 36], and may suggest some binding of the tracer at the bone marrow level. Consequently, blood-based imaging cannot be used for reliable evaluation of the bone marrow radiation dose; therefore, only image-based bone marrow dosimetry was performed in Part B.

The presence of bone metastases impacts the estimation of bone marrow absorbed dose by image-based techniques and its correlation with haematological toxicity [13]. The applied bone marrow absorbed-dose limit of 1.5 Gy, evaluated based on L_2_–L_4_ (or other vertebrae in case of tumour overlay in the lumbar vertebrae region or visible bone marrow involvement) over the first three cycles, was exceeded in three instances. In two of the patients, this was without any acute clinical manifestations and may have been caused by existing or appearing tumours, close to the region considered for bone marrow dosimetry (i.e., T_8_–T_10_ thoracic vertebrae). Furthermore, absorbed dose in these patients was lower when blood-based dosimetry was applied, however the most conservative approach for assessment of absorbed dose was chosen. For the third patient, treated with only one cycle of 6.0 GBq of [^177^Lu]Lu-satoreotide tetraxetan, the cumulative bone marrow absorbed dose reached 3.6 Gy without any lesions identified close to the ROI/VOI; this patient developed grade 3 thrombocytopenia, leading to study withdrawal. These findings highlight the difficulty of evaluating the absorbed dose to the normal bone marrow (independently of tumours or bone marrow involvement). This difficulty was also evidenced by the large variability of bone marrow ADCs in our study. Overall, dosimetry monitoring in the bone marrow during cycle 1 could not be achieved for 9/40 patients (22.5%), and the validity of the 1.5 Gy conservative threshold could not be verified; this threshold was exceeded in five patients in this study, though not all of these patients went on to experience haematological toxicity.

In the present study, grade 3/4 treatment-related haematological toxicity was reported in 14 patients (35.0%), all of which were transient and not associated with clinical manifestations such as bleedings or infections [24]. Acute haematological toxicity may be a dose-limiting factor for [^177^Lu]Lu-satoreotide tetraxetan, and for ^177^Lu-labelled somatostatin analogues in general. There is limited correlation between absorbed radiation doses to the bone marrow and development of haematologic toxicity [36–38]. Therefore, it is important to carefully monitor hematological parameters during treatment.

Compared to previously reported data on the SSTR agonist [^177^Lu]Lu-DOTA-TATE, [^177^Lu]Lu-satoreotide tetraxetan showed similar overall biodistribution and pharmacokinetic properties, with both showing rapid excretion through the kidneys following administration [32]. In addition, consistent with their specific binding to SSTR, both [^177^Lu]Lu-satoreotide tetraxetan and [^177^Lu]Lu-DOTA-TATE predominantly show uptake in tumours, followed by the kidneys, spleen, and liver [32]. [^177^Lu]Lu-DOTA-TATE has, however, been associated with a mean terminal blood half-life of 72 h [39, 40], which is lower than the 127-hour median (110-hour mean) terminal half-life reported with [^177^Lu]Lu-satoreotide tetraxetan at treatment cycle 1 in our study. The long terminal half-life of [^177^Lu]Lu-satoreotide tetraxetan is most likely the result of SSTR antagonists being retained by NETs for longer periods at the SSTR site and on circulating tumour cells in patients with NETs [22, 41, 42]. The prolonged retention of [^177^Lu]Lu-satoreotide tetraxetan within SSTR-expressing tissues is further supported by the different blood and organ uptake profiles observed here, as well as the presence of whole-body uptake at 6 days (Fig. 2).

The present study has some limitations, common to all phase I/II studies, including the small sample size and the heterogeneity of the study population. Dosimetry monitoring in the liver could only be performed for seven patients overall, as in most cases there were several lesions taking up [^177^Lu]Lu-satoreotide tetraxetan in the liver. There was only one patient in part B for whom a liver TIAC could be directly calculated for use in OLINDA/EXM analyses. Hence, the liver absorbed dose of [^177^Lu]Lu-satoreotide tetraxetan calculated by OLINDA/EXM may be an underestimation. Despite these shortcomings, this study is strengthened by the fact that all included data were collected prospectively and analysed centrally, and by the application of patient-specific dosimetry. Results from this study represent important preliminary findings regarding the dosimetric and pharmacokinetic properties of [^177^Lu]Lu-satoreotide tetraxetan. Further dedicated studies to better establish the relationship between dosimetry findings and the efficacy and safety of RLT, including [^177^Lu]Lu-satoreotide tetraxetan, are warranted.

## Conclusion

This completed biodistribution and dosimetry study, part of a phase I/II trial, demonstrated that [^177^Lu]Lu-satoreotide tetraxetan has a favourable pharmacokinetic and dosimetric profile, with high and prolonged tumour uptake, supporting its acceptable safety profile and promising efficacy.

### Electronic supplementary material

Below is the link to the electronic supplementary material.

Supplementary Material 1

## Data Availability

Qualified researchers may request access to patient-level study data that underlie the results reported in this publication. Additional relevant study documents, including the clinical study report, study protocol with any amendments, annotated case report form, statistical analysis plan and dataset specifications may also be made available. Patient level data will be anonymised, and study documents will be redacted to protect the privacy of study participants. The data that support the findings of this study are available from the corresponding author, Ben Pais (b.pais@ariceum-therapeutics.com), upon reasonable request.
