# Supplementary material for: Dosimetry and pharmacokinetics of [177Lu]Lu-satoreotide tetraxetan in patients with progressive neuroendocrine tumours
Source: Eur J Nucl Med Mol Imaging. 2024 Mar 26;51(8):2428–41. doi: 10.1007/s00259-024-06682-1 (PMC11178655; doi:10.1007/s00259-024-06682-1)
Supplement: Supplementary file 1 — Supplementary Material 1 [file 259_2024_6682_MOESM1_ESM.docx]

# **Original Article**

***European Journal of Nuclear Medicine and Molecular Imaging***

**Title:** Dosimetry and pharmacokinetics of [^177^Lu]Lu-satoreotide tetraxetan in patients with progressive neuroendocrine tumours

**Authors and Affiliations**

Seval Beykan Schürrle,^1*^ Uta Eberlein,^1*^ Catherine Ansquer,^2^ Jean-Mathieu Beauregard,^3^ Lucie Durand-Gasselin,^4^ Henning Grønbæk,^5^ Alexander Haug,^6^ Rodney J. Hicks,^7,8^ Nat P. Lenzo,^9,10^ Shaunak Navalkissoor,^11^ Guillaume P. Nicolas,^12^ Ben Pais,^13,14†^ Magali Volteau,^4^ Damian Wild,^12^ Alexander McEwan,^14‡^ Michael Lassmann^1^

^*^Shared first authorship

^1^Department of Nuclear Medicine, University Hospital Würzburg, Würzburg, Germany

^2^CHU Nantes, Nantes Université, Médecine Nucléaire, Nantes, France

^3^Department of Medical Imaging, CHU de Québec – Université Laval, Québec City, Quebec, Canada

^4^Ipsen Innovation, Les Ulis, France

^5^Department of Hepatology & Gastroenterology, ENETS Centre of Excellence, Aarhus University Hospital and Clinical Institute, Aarhus University, Aarhus, Denmark

^6^Department of Radiology and Nuclear Medicine, Medical University of Vienna, Vienna, Austria

^7^Department of Medicine, St Vincent’s Hospital, The University of Melbourne, Melbourne, Victoria, Australia

^8^Department of Medicine, Central Clinical School, the Alfred Hospital, Monash University, Melbourne, Victoria, Australia

^9^GenesisCare, East Fremantle, Western Australia, Australia

^10^Department of Medicine, Curtin University, Perth, Western Australia, Australia

^11^Neuroendocrine Tumour Unit, ENETS Centre of Excellence, Royal Free London NHS Foundation Trust, London, United Kingdom

^12^Division of Nuclear Medicine, ENETS Centre of Excellence, University Hospital Basel, Basel, Switzerland

^13^SRT-Biomedical B.V., Soest, Netherlands

^14^Ariceum Therapeutics GmbH, Berlin, Germany

^†^Former: Ipsen, Hoofddorp, Netherlands

^‡^Former: Ipsen Bioscience, Cambridge, Massachusetts, United States

**Correspondence:** Ben Pais, [b.pais@ariceum-therapeutics.com](mailto:b.pais@ariceum-therapeutics.com)

**Manuscript details:** 6,110/6,000 (including references); 3 Tables; 3 Figures.

# **Acknowledgments**

The authors would like to thank all patients involved in the study, their caregivers, care team, investigators, and research staff in the participating institutions. The authors would like to thank Peter Iversen, MD, PhD, Aarhus University Hospital, Aarhus, Denmark, for his involvement in the study. The authors would like to thank Thomas Rohban, MD, and Magalie El Hajj, PharmD, from Partner 4 Health (Paris, France) and Patrick Cox, BSc, Jessica A. Buttress, PhD, and Erin Clarkson, BSc, from Costello Medical (London and Cambridge, UK) for providing medical writing support, which was sponsored by Ipsen in accordance with Good Publication Practice guidelines.

# **Supplementary Information**

**Plain Language Summary**

Neuroendocrine tumours (NETs) are rare cancers that grow from cells that release hormones. NETs can form in many different organs of the body. Radioligand therapy (RLT) is a type of cancer treatment which delivers radiation to cancer cells inside the body. RLT is designed to specifically treat tumours, such that a high dose of radiation is given to cancer cells, while little radiation is given to the healthy surrounding cells.

[^177^Lu]Lu-satoreotide tetraxetan is a new type of RLT which may be an effective treatment for patients with NETs. Because radiation is harmful to both cancer and normal cells, this study investigated how much radiation [^177^Lu]Lu-satoreotide tetraxetan delivered to different parts of the body.

Forty patients were treated with varying amounts and strengths of [^177^Lu]Lu-satoreotide tetraxetan. The amount of radiation that was delivered to the cancer cells and different organs in the body was then measured.

The results of the study showed that [^177^Lu]Lu-satoreotide tetraxetan is safe for use in patients with NETs, when the amount of radioactivity (which helps deliver the treatment to the cancer cells) is limited, to reduce the amount of radiation to healthy organs. As [^177^Lu]Lu-satoreotide tetraxetan is designed to specifically treat cancer cells, the treatment was taken up by cancerous tumours more than other healthy organs, and it remained in the tumours for a long time. The amount of radiation given to healthy organs (such as the bone marrow, kidneys, liver, and spleen) was within safe limits in most patients in this study. [^177^Lu]Lu-satoreotide tetraxetan was mainly removed from the body by the kidneys.

These findings will help researchers to design future studies on [^177^Lu]Lu-satoreotide tetraxetan, supporting the development of a new, effective treatment option for patients with NETs.

**Methods**

**Image acquisition**

All images were acquired with a 20% energy window around the main photopeak of ^177^Lu (208 keV). For scatter correction, the triple-energy window method was applied based on the recommendation of the Committee on Medical Internal Radiation Dose [1]. Whole-body images were acquired with a scanning time of 20 cm/min in a 256 × 1,024 matrix. SPECT imaging was performed with a 15-second frame duration for 120–128 projections, applying 60–64 views per head over 180° in a 128 × 128 matrix size. Co-registered CTs were acquired in low-dose technique, with a 5-mm slice thickness and a 0.99-mm pixel size (130 kVp, 30 mA), and used to generate an attenuation map. SPECT images were iteratively reconstructed, with a minimum of 36 updates, using the ordered subset expectation maximisation (OSEM) algorithm, with collimator depth-dependent three-dimensional resolution recovery (if available) and attenuation correction taken from the µ-maps obtained by the system software from the CT images. No filtering was applied. A syringe containing a known ^177^Lu activity (approximately 20 MBq) was added in the field of view of the whole-body images to track the stability of the gamma cameras during each therapy cycle, for quality control purposes. For part B, an additional syringe measurement at a single timepoint was performed on SPECT/CT acquisitions.

**Delineation and [^177^Lu]Lu-satoreotide tetraxetan activity quantification**

For evaluated organs, regions/volumes of interest (ROIs/VOIs) were drawn manually over the whole organs of interest (whole body, left and right kidneys, liver, spleen, and bone marrow), using the NUKDOS software for planar whole-body scans and the SPECT/CT acquired in part A [2]. For all SPECT/CT images acquired in part B, VOIs were drawn in a SIEMENS E.Soft workstation. For part A planar whole-body scans, an additional irregularly shaped ROI was placed around each target ROI for background subtraction. The corresponding organ volumes were taken from the corresponding CT images, and organ masses were derived by applying a tissue density of 1.0 g/cm^3^. For the bone marrow, ROIs/VOIs were drawn on the L_2_–L_4_ lumbar vertebrae [3, 4], assuming that this location is reflective of the absorbed dose to the overall bone marrow. In case of overlapping activities in the L_2_–L_4_ vertebrae on the planar and SPECT/CT scans associated with lesions or bone marrow involvement, other vertebrae were taken as surrogates for the bone marrow. If no other vertebrae were available, no ROI or VOI was drawn. For the kidneys, ROIs/VOIs were drawn over the renal cortex, or, in case the cortex could not be separated, over the whole kidney.

All ROIs/VOIs were then used for activity quantification. Using the NUKDOS software, the time-integrated activity coefficients (TIACs) for a given source volume were computed by integrating the time-activity curves.

**Image calibration**

To ensure comparability of the gamma counter results and the image quantification of the SPECT/CT images at each study centre, three low-activity samples were centrally prepared and shipped to the study sites. The traceability of the activity in the samples was determined by measuring each sample in a high-purity germanium detector (Canberra GmBH, Germany) at the central site. The counting efficiency of the detector was ascertained by using standards traceable to the National Institute of Standards and Technology and the National Physical Laboratory, United Kingdom. In principle, the calibration process setup was as follows:

1. On-site preparation of two different dilutions of ^177^Lu activity, determined in a calibrated activity meter, for filling a phantom for SPECT/CT imaging and for calibrating the well counter.
2. Determination of the image calibration factor (ICF) by imaging a phantom with SPECT/CT with standardised acquisition and reconstruction settings.
3. Determination of the well counter calibration factor.
4. Measurement of three calibrated low-activity well counter samples provided by the central site.
5. Comparison of the activity values of the three samples to the values provided by the central site. If the deviations were >10%, the procedure was to be repeated until an acceptable deviation was reached.
6. Well counter measurement of a low-activity aliquot of the stock solution for filling the phantom. If the value of the diluted activity and the value measured in the gamma counter deviated by >10%, the procedure was to be repeated until an acceptable deviation was reached.
7. Comparison of the vendor-specific ICF to the ICF of the calibration site (for SIEMENS SPECT/CT systems only). If the deviation was <5%, the site-specific ICF was deemed acceptable.

**Dosimetry**

Patient-specific dosimetry calculations were performed according to European Association of Nuclear Medicine Dosimetry Guidance [5] after each treatment cycle in both parts A and B, using the absorbed dose calculation features of the NUKDOS software [2] (i.e., integration of the time-activity curves, calculation of the absorbed dose). Model-based dosimetry calculations were only performed after the first [^177^Lu]Lu-satoreotide tetraxetan cycle and for patients in part B. To generate organ-specific absorbed doses, TIACs were used as input data in the Organ Level INternal Dose Assessment/EXponential Modelling (OLINDA/EXM) software version 1.0, which was granted Food and Drug Administration 510(k) clearance in June 2004. The following settings were applied: ^177^Lu isotope, no bladder voiding model, adult male or female phantom. As the OLINDA/EXM software makes no distinction between the right and left kidneys, their TIACs were summed. In case biokinetic data were available for one kidney only, the kidney TIAC value was doubled. For the bone marrow, the patient-specific mass of the analysed vertebrae was used for upscaling image-based TIAC values according to the OLINDA/EXM phantom gender-specific masses (m_Bone marrow_=1,120 g for males and 1,300 g for females):

$${TIAC}_{Bone marrow}= {TIAC}_{L2-L4}\frac{m_{Bone marrow}}{m_{L2-L4}}$$

The kidney, spleen, liver, and bone marrow TIACs were subtracted from the patient’s whole-body TIAC to obtain the TIAC of the remainder of the body.

**Results**

**Pharmacokinetics**

Kidneys were the main route of elimination of [^177^Lu]Lu-satoreotide tetraxetan. The median radioactivity excreted via urine within 48 hours of administration of the first treatment cycle ranged between 2.6–3.0 GBq across groups in patients treated with 4.5 GBq, and was 3.4 GBq in those treated with 6 GBq (corresponding to approximately 57–66% and 57% of the administered activity of [^177^Lu]Lu-satoreotide tetraxetan, respectively). Following the first treatment cycle, [^177^Lu]Lu-satoreotide tetraxetan rapidly reached its peak in the blood (median blood uptake at 1–5 minutes of 3.03% administered activity/L), before exponentially decreasing with a median terminal blood half-life of 127 hours (range: 38.6–160.0 hours; mean ± SD: 110.0 ± 50.1) and a mean extrapolated area under the concentration-time curve (AUC_0–infinity_) of 742.0 ± 303.0 MBq.h/L. Subsequent cycles exhibited similar characteristics.

**Table S1.** Overview of [^177^Lu]Lu-satoreotide tetraxetan treatment.

|  | **Part A (N=15)** | **Cohort 1 of part B (N=6)** | **Cohort 3 of part B (N=9)** | **Cohort 6 of part B (N=10)** | **Part B (N=25)** | **Total (N=40)** |
| --- | --- | --- | --- | --- | --- | --- |
| Median cumulative administered activity (range) (GBq) | 13.1*  (10.3–13.5) | 9.1  (4.3–15.4) | 13.0  (8.5–17.6) | 13.1  (4.2–20.8) | 12.9  (4.2–20.8) | 13.0  (4.2–20.8) |
| **Number (%) of patients with a total number of cycles** | | | | | | |
| 1 cycle | 0 | 2 (33.3) | 0 | 2 (20.0) | 4 (16.0) | 4 (10.0) |
| 2 cycles | 0 | 3 (50.0) | 0 | 2 (20.0) | 5 (20.0) | 5 (12.5) |
| 3 cycles | 15 (100) | 1 (16.7) | 5 (55.6) | 4 (40.0) | 10 (40.0) | 25 (62.5) |
| 4 cycles | - | 0 | 4 (44.4) | 1 (10.0) | 5 (20.0) | 5 (12.5) |
| 5 cycles | - | 0 | 0 | 1 (10.0) | 1 (4.0) | 1 (2.5) |

Overall study population (N=40). Data are presented as n (%), unless otherwise specified. *n=14.

**Table S2**. Absorbed dose coefficients of [^177^Lu]Lu-satoreotide tetraxetan at the first treatment cycle in all lesions by initial diagnosis.

|  | **Initial diagnosis** | | |
| --- | --- | --- | --- |
| **Overall specific absorbed dose coefficient, Gy/GBq** | **GEP-NET** | **Lung NET** | **Pheochromocytoma + paraganglioma** |
| N | 23 | 6 | 4 |
| Median | 5.8 | 5.1 | 2.1 |
| Range | 0.6–83.3 | 2.1–28.2 | 0.4–4.9 |

Per protocol dosimetry analysis set (N=36). (GEP)-NET: (gastroenteropancreatic)-neuroendocrine tumour.

**Fig. S1.** Absorbed dose coefficients of [^177^Lu]Lu-satoreotide tetraxetan for evaluated organs after administration of the first treatment cycle in parts A and B.


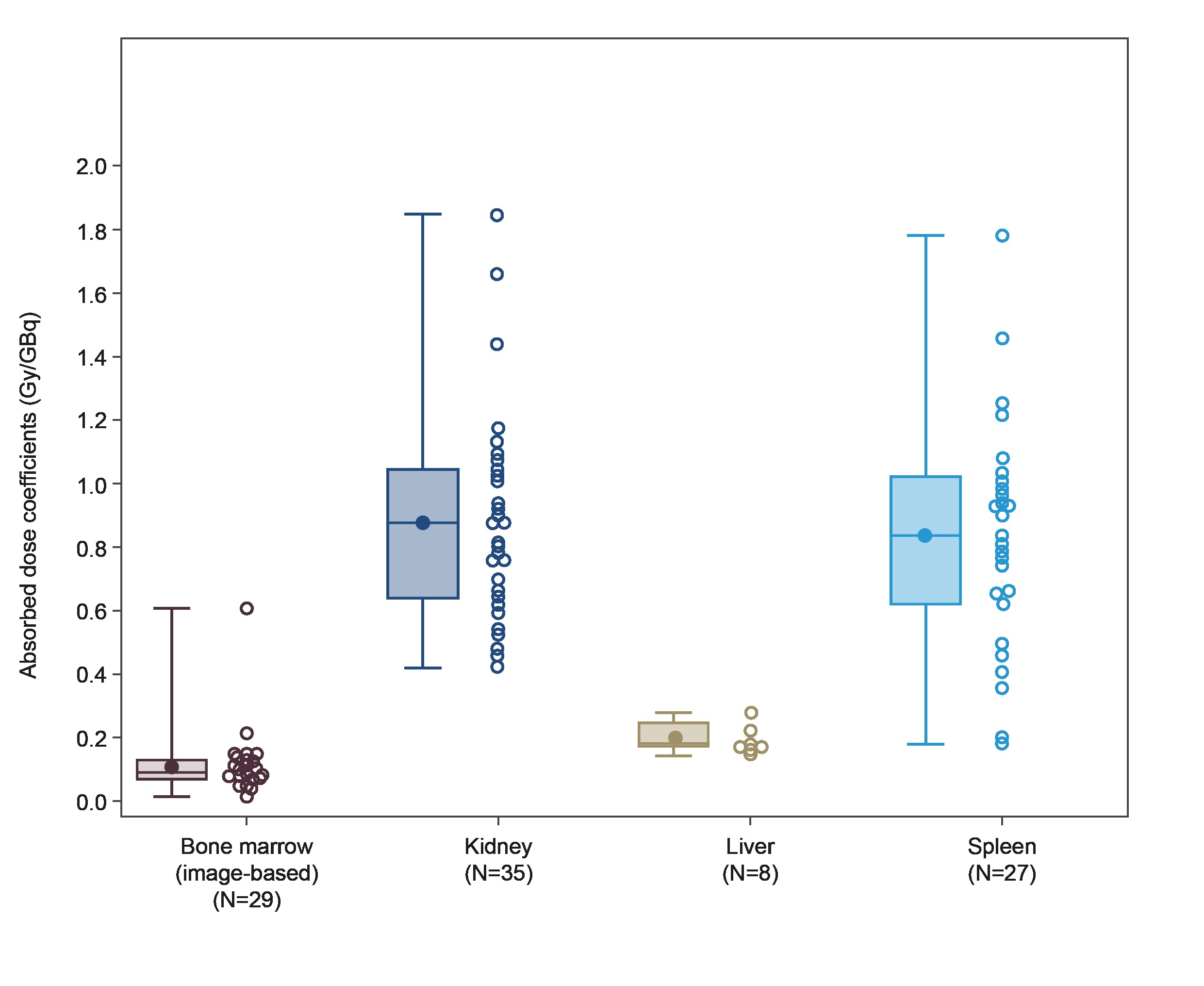
Data are presented as mean, median (minimum, Q1, Q2, Q3, maximum) for parts A and B in box plot; scatter plots represent individual patient data for parts A and B. The first treatment cycle corresponds to 4.5 GBq/300 µg of [^177^Lu]Lu-satoreotide tetraxetan in part A and cohorts 3 and 6 of part B and 6.0 GBq/300 µg in cohort 1 of part B; in cohort 1 of part B, administered activity was reduced to 4.5 GBq after the first three patients following the recommendation of the Data Review Board. Q1: quartile 1; Q2: quartile 2; Q3: quartile 3.

1. Ljungberg, M., et al., *MIRD Pamphlet No. 26: Joint EANM/MIRD Guidelines for Quantitative 177Lu SPECT Applied for Dosimetry of Radiopharmaceutical Therapy.* Journal of Nuclear Medicine, 2016. **57**(1): p. 151–162 DOI: 10.2967/jnumed.115.159012.

2. Kletting, P., et al., *The NUKDOS software for treatment planning in molecular radiotherapy.* Zeitschrift für Medizinische Physik, 2015. **25**(3): p. 264-274 DOI: <https://doi.org/10.1016/j.zemedi.2015.01.001>.

3. Blakkisrud, J., et al., *Red Marrow–Absorbed Dose for Non-Hodgkin Lymphoma Patients Treated with &lt;sup&gt;177&lt;/sup&gt;Lu-Lilotomab Satetraxetan, a Novel Anti-CD37 Antibody–Radionuclide Conjugate.* Journal of Nuclear Medicine, 2017. **58**(1): p. 55 DOI: 10.2967/jnumed.116.180471.

4. Ferrer, L., et al., *Three methods assessing red marrow dosimetry in lymphoma patients treated with radioimmunotherapy.* Cancer, 2010. **116**(S4): p. 1093-1100 DOI: <https://doi.org/10.1002/cncr.24797>.

5. Lassmann, M., et al., *EANM Dosimetry Committee guidance document: good practice of clinical dosimetry reporting.* European Journal of Nuclear Medicine and Molecular Imaging, 2011. **38**(1): p. 192-200 DOI: 10.1007/s00259-010-1549-3.
